# *Mycobacterium tuberculosis* polyclonal infections through treatment and recurrence

**DOI:** 10.1371/journal.pone.0237345

**Published:** 2020-08-19

**Authors:** Pooja Pandey, Anuj K. Bhatnagar, Anant Mohan, Kuldeep S. Sachdeva, Jyotish C. Samantaray, Randeep Guleria, Urvashi B. Singh

**Affiliations:** 1 Department of Microbiology, All India Institute of Medical Sciences, New Delhi, India; 2 Rajan Babu Institute for Pulmonary Medicine and Tuberculosis, Delhi, India; 3 Department of Pulmonary Medicine and Sleep Disorders, All India Institute of Medical Sciences, New Delhi, India; 4 Central TB Division, Government of India, New Delhi, India; Institut de Pharmacologie et de Biologie Structurale, FRANCE

## Abstract

**Background:**

Mixed/polyclonal infections due to different genotypes are reported in Tuberculosis. The current study was designed to understand the fate of mixed infections during the course of treatment and follow-up and its role in disease pathogenesis.

**Methods:**

Sputum samples were collected on 0,1,2,3,6,12 and 24 months from 157 treatment-naïve patients, cultures subjected to Drug-Susceptibility-testing (MGIT 960), spoligotyping, MIRU-VNTR and SNP genotyping. All isolated colonies on thin layer agar (7H11) were subjected to spoligotyping.

**Findings:**

One thirty three baseline cultures were positive (133/157, 84.7%), 43(32.3%) had mixture of genotypes. Twenty-four of these patients (55.8%) showed change in genotype while six showed different drug-susceptibility patterns while on treatment. Twenty-three (53.5%) patients with polyclonal infections showed resistance to at least one drug compared to 10/90 (11.1%) monoclonal infections (P<0.0001). Eight patients had recurrent TB, two with a new genotype and two with altered phenotypic DST.

**Conclusions:**

The coexistence of different genotypes and change of genotypes during the same disease episode, while on treatment, confirms constancy of polyclonal infections. The composition of the mixture of genotypes and the relative predominance may be missed by culture due to its limit of detection. Polyclonal infections in TB could be a rule rather than exception and challenges the age-old dogma of reactivation/reinfection.

## Introduction

*Mycobacterium tuberculosis* (Mtb) infects and kills millions of people worldwide. [1] Traditionally, Tuberculosis (TB) is understood to be caused by a single genotype of Mtb, but recent reports of mixed infections challenge the old dogma.

Comas et al., 2013 proposed and confirmed that congruence exists between the MTBC phylogeny and human mitochondrial genomes. [2] On the basis of which *it was propounded that Mycobacterium tuberculosis* complex (MTBC) has clonally expanded from a progenitor population which might have arisen 70,000 years ago and it spread across with human migration. [2] A large proportion of acquired mutations in MTBC lead to phenotypic differences and diversity. Multiple TB strains infecting patients, could have impact on accurate disease diagnosis, affect treatment, and possibly control of TB. [3,4] Polyclonal infections may be associated with Hetero-resistance. [5–8]. Zetola et al., 2014, showed that false-negative results using Gene-Xpert MTB/RIF were significantly associated with mixed infections and that it fails to detect rifampicin (RIF)resistance in vitro when resistant sub-populations accounted for <90%. [6,9]

The advent of newer standardized TB strain-typing methodologies has provided us an opportunity to re-evaluate the epidemiology of this ancient scourge. It is reported that polyclonal infections may remain occult during the initial treatment and hence may affect the outcome. [7] The prevalence of mixed TB infection remains uncertain and estimates from previous studies are likely underestimated. [8] Various studies on mixed infections in TB have shown that 10–20% of the patients can be simultaneously infected with multiple strains at one point in time [3, 10–13], strongly suggesting that mixed infections are common.

The current study was designed to not only detect baseline prevalence of mixed TB infection but primarily to study the fate of polyclonal infections in patients initiated on treatment.

## Materials and methods

The study was conducted at the Tuberculosis Division, Department of Microbiology, All India Institute of Medical Sciences (AIIMS), New Delhi, India. All methods were carried in accordance with relevant guidelines and regulations. A written informed consent was obtained from the participants. The study was ethically approved by the AIIMS Ethics Committee (IESC/T221 92/01.02.2013). Drug naïve category I patients were enrolled before initiating anti-tubercular therapy and were formally followed for two years. [14] The patient participation was voluntary, and compliance to medication was reiterated by treating physician and trained medical staff. Sputum samples were collected at 0,1,2,3,6,12 and 24 months from all patients.

### Smear preparation, staining and culture

One spot and one early morning sample was collected and processed using NALC-NaoH method.[15] Samples were inoculated into liquid culture (MGIT, Becton Dickinson, Sparks, MD, USA) solid culture (LJ, 7H11 agar).

#### AFB smears

Smears were stained by the Ziehl Neelsen method. [16] The results were quantified in accordance with RNTCP standards. [16]

#### Culture

Samples were inoculated into MGIT 960 automated isolation system (Becton Dickinson, Sparks, MD, USA) according to manufacturer’s instructions; All positive cultures were confirmed as *Mycobacterium tuberculosis* using TBc Identification Test (TBc ID, Becton Dickinson, Sparks, MD, USA). [17] Biochemical identification and speciation was done for pure cultures. Cross contamination between samples was avoided by using processing controls.

The phenotypic drug susceptibility testing (DST) was performed for all positive liquid cultures using MGIT 960. DST for RIF and INH was performed with the MGIT 960 system, using the manufacturer’s protocol. [18–20]

#### Lowenstein Jensen medium (LJ)

Lowenstein Jensen medium was prepared using the standard protocol. [16] 500μl of the decontaminated samples was inoculated in the LJ medium. DNA was extracted from positive LJ slopes using the heat lysis method.

### Culture methods for detection of mixed infection

#### Thin layer agar (TLA)

The TLA technique was used for the early diagnosis of TB by detection of micro-colonies of mycobacteria. [21] TLA (7H11 agar) was prepared using standard protocols. [22] Five to 12 colonies from 7H11 TLA were initially suspended in sterile 7H9 medium with OADC. These tubes were incubated at 37°C and observed for growth weekly till 7 weeks. The positive cultures were inoculated on LJ medium. DNA extraction was done from all the positive LJ cultures. All samples showing contamination were re-processed and re-inoculated in the respective medium.

### Molecular genotyping

#### Spoligotyping

Spoligotyping was performed according to a standard protocol [[23] Classification of the spoligotype family was based on the international database SITVIT [24].

#### MIRU-VNTR

Standard 24-loci MIRU-VNTR typing was done for all VNTR loci using previously defined sets of primers and conditions. [25] The number of MIRU-VNTR repeats was determined by comparing the predicted allelic size to the H37Rv genotype using 24 MIRU-VNTR standard loci and agarose gel electrophoresis that was described previously.[25]

#### SNP genotyping for classifying into main phylogenetic lineages

SNP genotyping by Taq Man real-time PCR assays was done with the objective to classify the strains into lineages using the standard protocols. [26] Reactions were run in a Step One Plus thermocycler (Applied Biosystems; 60°C 30 sec; 95°C 10 min; 95°C 15 sec and 60°C 1 min for 40 cycles; 60°C 30 sec) and fluorescence intensity in the VIC and FAM channels measured at the end of every cycle. Results were analyzed with Step One software (Applied Biosystems) and alleles called with the default algorithm.

#### Definition of mixed infections by spoligotyping, real time based SNP genotyping and VNTR typing

Spoligotyping defined mixed infections by the presence of different lineages in a sample. MIRU-VNTR typing uses presence of double alleles in two or more VNTR loci for detection of mixed infections. [27] SNP-genotyping defined mixed infection if two SNP’s were positive at the same time. [26] Single colony spoligotyping was also used to detect mixed infections.

### Statistical methods

All data were analyzed using STATA statistical software version 12.1 (Stata Corp LP, College Station, TX, USA). A Fisher exact test was performed to determine the association of polyclonal disease with different risk factors. A p-value of <0.05 was regarded as significant.

## Results

### Study population

One hundred fifty-seven patients with complaints of cough, fever, chest pain, expectoration or hemoptysis but no past history of TB treatment was enrolled for the study. Following diagnosis, all patients were put on RNTCP recommended treatment. [14] Six of 157 patients enrolled were diagnosed with Rifampicin resistance and initiated on multi-drug resistance (MDR) treatment regimen. [14] Patients were followed formally for a period of two years, and compliance to treatment ensured, none were lost to follow up; eight patients had recurrence. [14, 28] Females constituted 32.5% of the patients enrolled, and males 67.5%; 12.1% stayed in rural setting, 87.9% in urban; 21% had family history of TB; 52.2% had smoking history; 24.8% were addicted to alcohol; 55.4% were BCG vaccinated. [S1 Table]

### Baseline cultures and DST

Baseline cultures were positive in 133 samples. All the cultures were confirmed as Mtb and none were Non-tubercular mycobacteria. [29] Thirty-seven (27.8%) patients were culture positive for baseline and early time points (i.e. 1,2 months), cultures at subsequent time points [6, 12 and 24 months] were negative. Drug susceptibility testing (DST) was done on baseline cultures. DST identified 100 (75.2%) isolates as sensitive to all 4 drugs tested, 6/133(4.5%) as MDR, 27 (20.3%) resistant to one/more drugs [24(18%) mono-resistant, 3(2.3%) resistant to 2 drugs and no triple drug resistance] [S2 Table]

### Characterization of isolates using spoligotyping, MIRU-VNTR typing, SNP genotyping

DNA extracted from Lowenstein Jensen (LJ) medium was subjected to three typing methods namely spoligotyping, MIRU-VNTR typing, Single Nucleotide Polymorphism (SNP) genotyping. Discrepant results by different methods, were repeated with the same DNA, to check reproducibility. Spoligotyping results revealed CAS lineage as most predominant genotype (48.8%) followed by EAI (25.6%), BEIJING (5.3%), T family (5.3%), MANU (1.5%), LAM (1.5%), H (1.5%), and X (0.7%) family. Thirteen (9.8%) strains were unstipulated and were termed orphan strains (Or) [defined as a unique spoligotype pattern not described in the SPOLDB4 database] [S3 Table] [24]

MIRU-VNTR also found CAS lineage (49.6%) as most predominant, followed by EAI (25.6%), BEIJING (5.3%), T family (5.3%), MANU (1.5%), LAM (0.7%), H (1.5%), and X family (1.5%). Twelve (9.0%) strains were unstipulated and were termed orphan strains (Or). There was no appreciable difference between the two methods; MIRU-VNTR additionally identified one isolate as CAS1_DELHI type. [S1 and S2 Figs]

SNP genotyping demonstrated four different *M*.*tb* lineages (Lineages 5 and 6 (*M*. *africanum*, West African lineages) have not been reported in our region). The most frequent Lineage 3(includes CAS/Delhi), with 65 isolates (48.9%) was followed by Lineage 1 (Indo-Oceanic Lineage, includes EAI) with 36 isolates (27%). Thirteen isolates (9.8%) were Lineage 4 (Euro-American Lineage), and 7 isolates (5.3%) Lineage 2 (East-Asian lineage, includes Beijing genotype). Twelve isolates were orphans and were grouped as Orphans by other methods as well. [S4 Table]

### Identification of mixed infections by MIRU-VNTR typing and SNP genotyping

Thirty-seven of 133 isolates were found to harbor mixed infections, using the three genotyping methods. VNTR typing has been shown to be sensitive in the detection of mixed infections by revealing double alleles. Twenty-nine isolates revealed double alleles at two or more loci, while 8 isolates were detected with double alleles at one locus, suggesting possible micro-evolution but these isolates showed mixed infection by both spoligotyping and SNP genotyping. [S5 Table] [27]. Another 3 isolates had double alleles at one locus, and none of the other typing methods demonstrated mixed infection. These strains could possibly represent microevolution. One strain detected mixed infection by MIRU-VNTR, which was missed by Spoligotyping and SNP genotyping. Cultures detected as mixed infection by at least two methods were considered Polyclonal.[27] None of the follow up samples gave evidence for microevolution. SNP genotyping was used for the first time for detection of Polyclonal infections.

### Single colony spoligotyping for polyclonal infection detection

**Fig 1:**
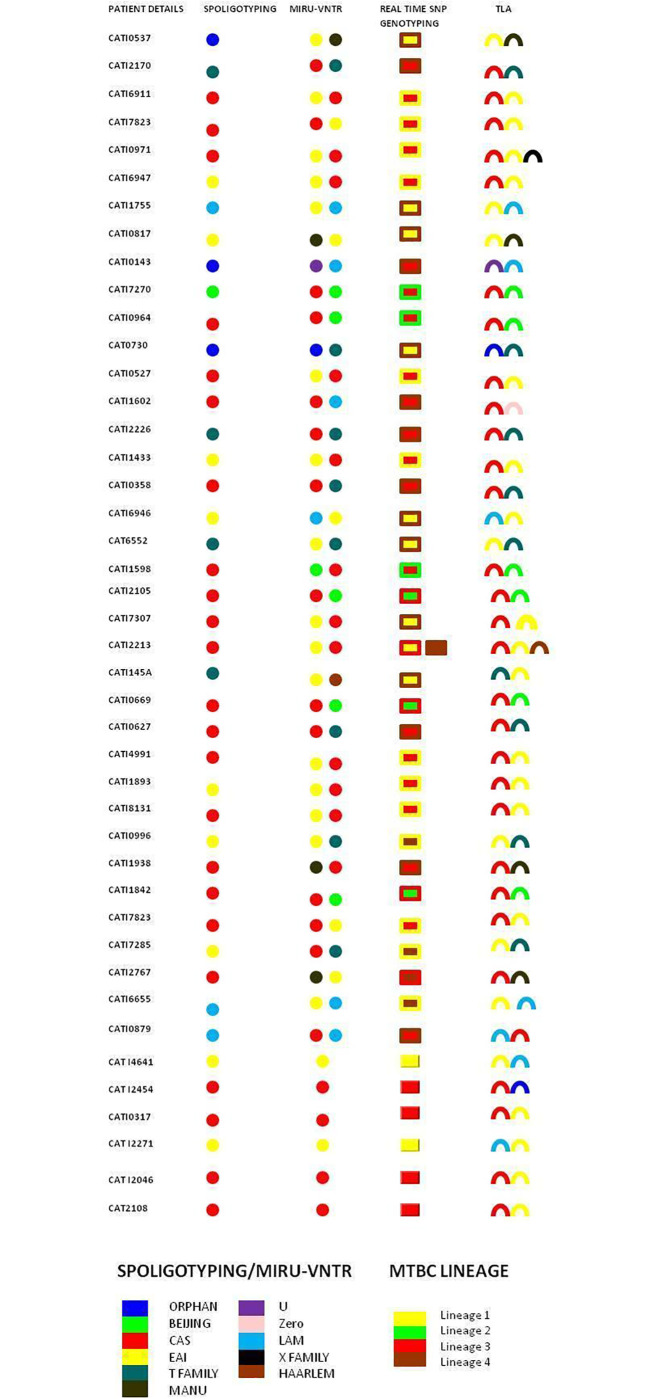
Baseline culture spoligotyping along with results of MIRU-VNTR, SNP typing and single colony spoligotyping (from TLA) used for detection of polyclonal infections. [17, 54–55] Twenty-nine samples gave concordant results by different genotyping techniques and Single Colony Spoligotyping (from TLA). Three samples revealed Orphan in baseline Spoligotyping but MIRU-VNTR and SNP genotyping identified mixed infection. Five cultures revealed discordant genotyping data on comparing three typing techniques and Single Colony Spoligotyping (from TLA). Single Colony Spoligotyping identified six samples with mixed infection, though missed by other genotyping techniques.

Analysis of all single colonies (5 to 12 colonies were isolated from different samples on TLA) by spoligotyping showed the coexistence of different genotypes in individual sputum samples. Polyclonal infections were detected in 43/133 (32.3%) patients, while baseline cultures using all the three typing methods namely spoligotyping, MIRU-VNTR and SNP genotyping could identify mixed infections in 37/133 (27.8%) patients. [Fig 1]

### Polyclonal infection in serial cultures at different time points

**Fig 2:**
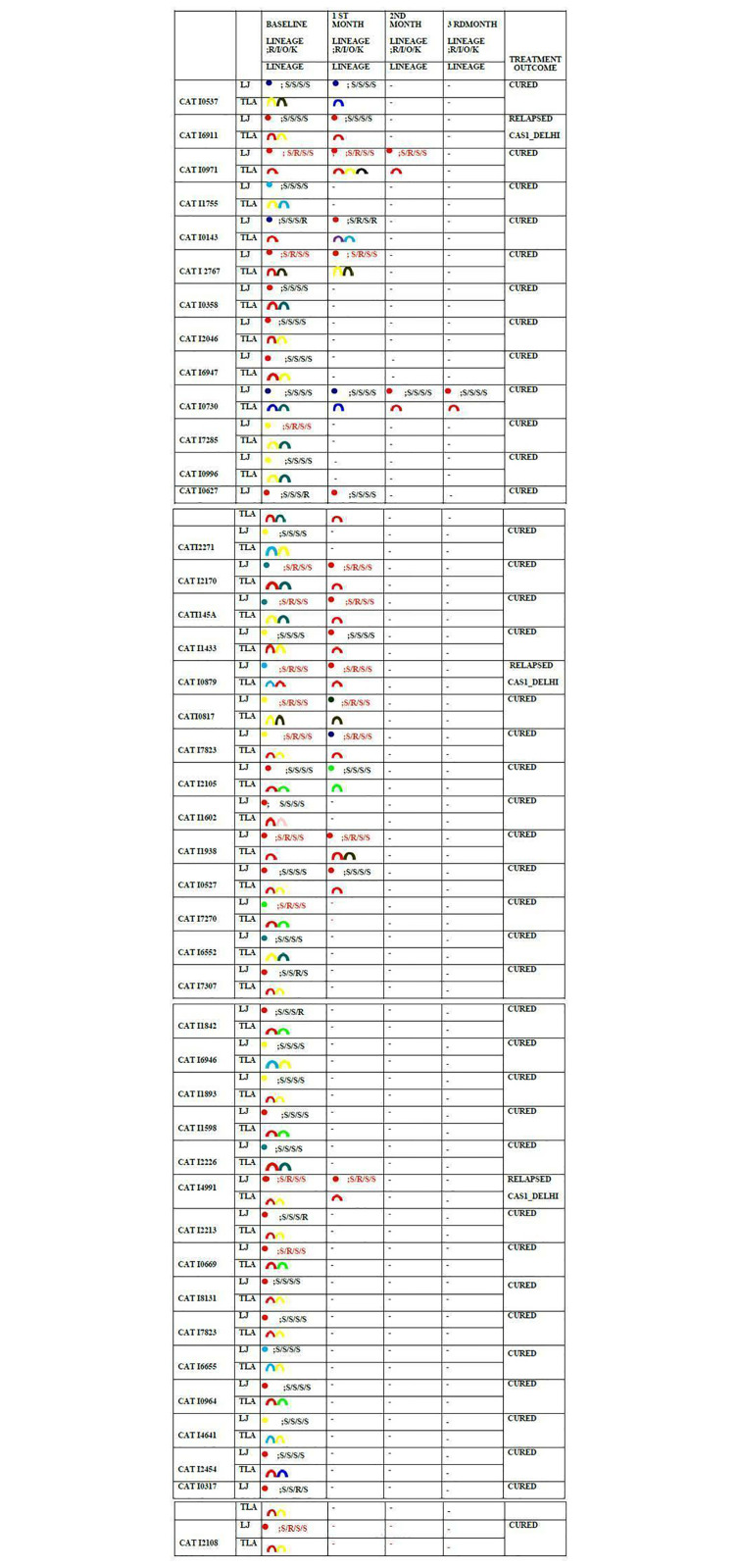
Serial cultures during patient follow-up on treatment, showing baseline culture spoligotyping results along with single colony spoligotyping (TLA) and phenotypic drug susceptibility profiles during different time points for patients with polyclonal infections. Baseline spoligotyping results did not always match with the results from single colony spoligotyping. A complete change of genotype was noted in the 1^st^ month in several patients. A change in the phenotypic drug susceptibility profile was also observed. CAS genotype constituted an increasing proportion of follow-up samples, and was the only genotype grown in 2^nd^ and 3^rd^ month cultures. [14, 15].

Twenty-four of 37 (64.9%) patients with serial cultures positive, showed different genotypes at baseline and at different time points by spoligotyping. The genotype in later time point/s in serial cultures was not always from the same lineages as at baseline. Two of 43 samples showed novel unrelated genotypes from another lineage. Six of the 24 (25%) patients with different genotypes, showed different drug susceptibility patterns. [Fig 2]

### Association of polyclonal infection with disease

Polyclonal infections were significantly more likely to occur in patients with high bacillary load, such as those with 2+ or 3+ smear grading, (*P<0*.*0001*). Polyclonality was significantly associated with any form of drug resistance (P<0.0001). Polyclonal infection was found to be associated with cavitary disease when compared to non-cavitary form. BCG vaccination seemed to protect against polyclonal infections (P<0.0001). However, there were no significant associations between the probability of having a polyclonal infection and age, sex, treatment outcome, and smoking. [Table 1]

**Table 1:** Risk factors associated with polyclonal disease. (P value is calculated by Fisher’s exact test. A p-value of <0.05 was regarded as significant).

Characteristic		No. [%) of patients		P value
		Total	Mixed infections	
**Total**		157	37	
**Sex**	Male	106	23	0.5644
	Female	51	14	
**Age [yr)**	15–34	119	28	0.6799
	35–60	38	11	
**Smear grade**	Negative or scanty	36	1	P<0.0001
	1+	62	6	
	2+	40	17	
	3+	19	13	
**Treatment outcome**	Favourable	149	35	1.0000
	Unfavourable	8	2	
**Smoker**	Yes	75	15	0.4677
	No	82	22	
**Family TB history**	Yes	33	12	0.1923
	No	124	25	
**BCG**	Yes	87	7	P<0.0001
	No	70	30	
**Abnormalities of X ray**	Cavitary	68	24	0.0272
	Non-cavitary	89	13	
**Alcohol**	Alcoholic	118	21	0.0410
	Non-alcoholic	39	16	

### Polyclonality vs drug resistance

**Fig 3:**
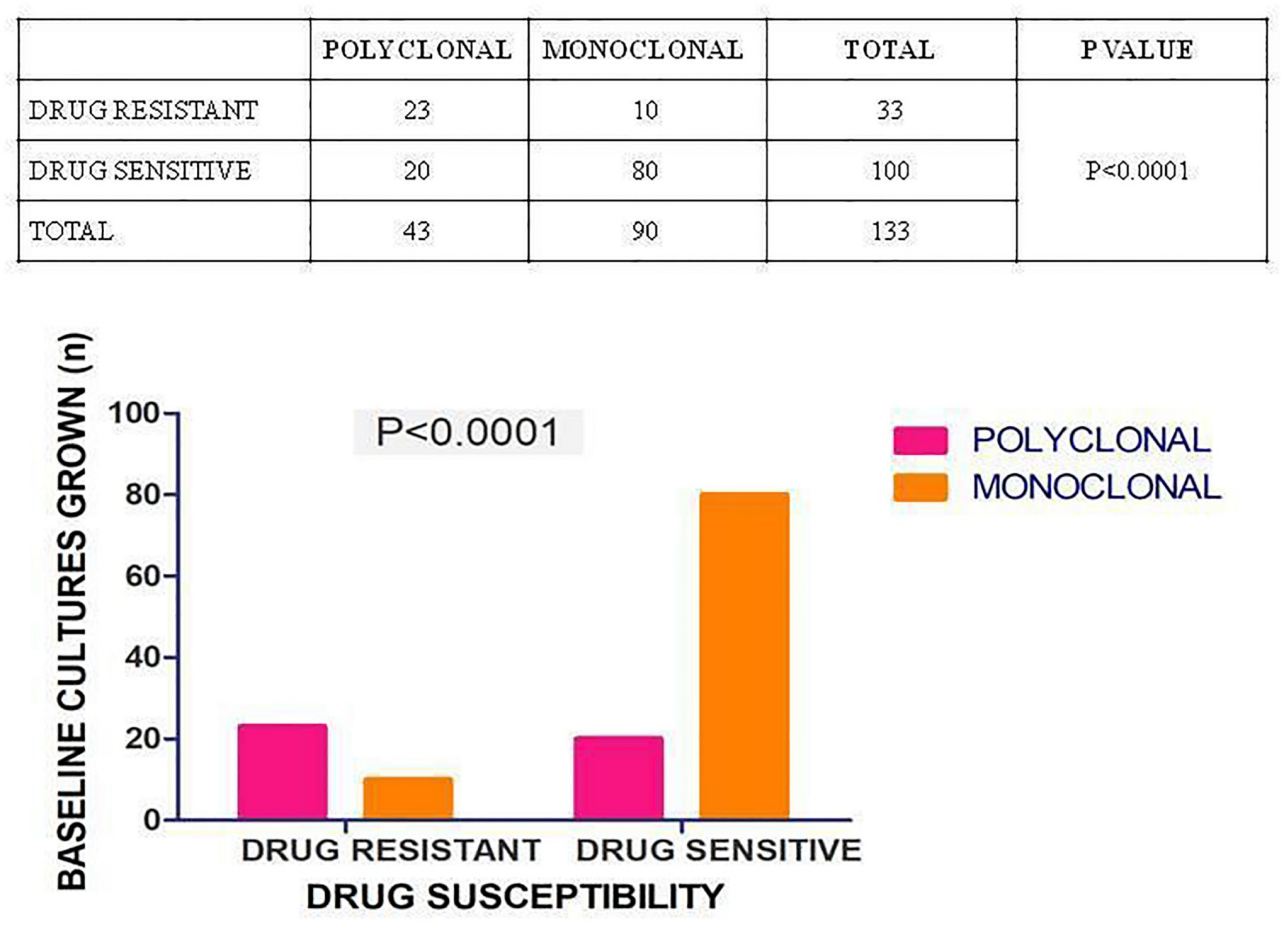
Association of polyclonal infections with drug resistant form of disease. Drug susceptibility test was performed on all baseline positive cultures (133). P value was calculated by Fisher’s exact test for determining the association of polyclonal disease with resistance. A p-value of <0.0001 was regarded as significant.

Twenty three of 33 (69.7%) patients exhibiting any drug resistance, showed presence of polyclonal infections and 23/43 (53.5%) patients harboring polyclonal infections showed presence of any drug resistance. Statistical analysis showed strong association of polyclonality with drug resistance. [Fig 3]

### Contribution of CAS lineage to polyclonality and drug resistance

**Fig 4:**
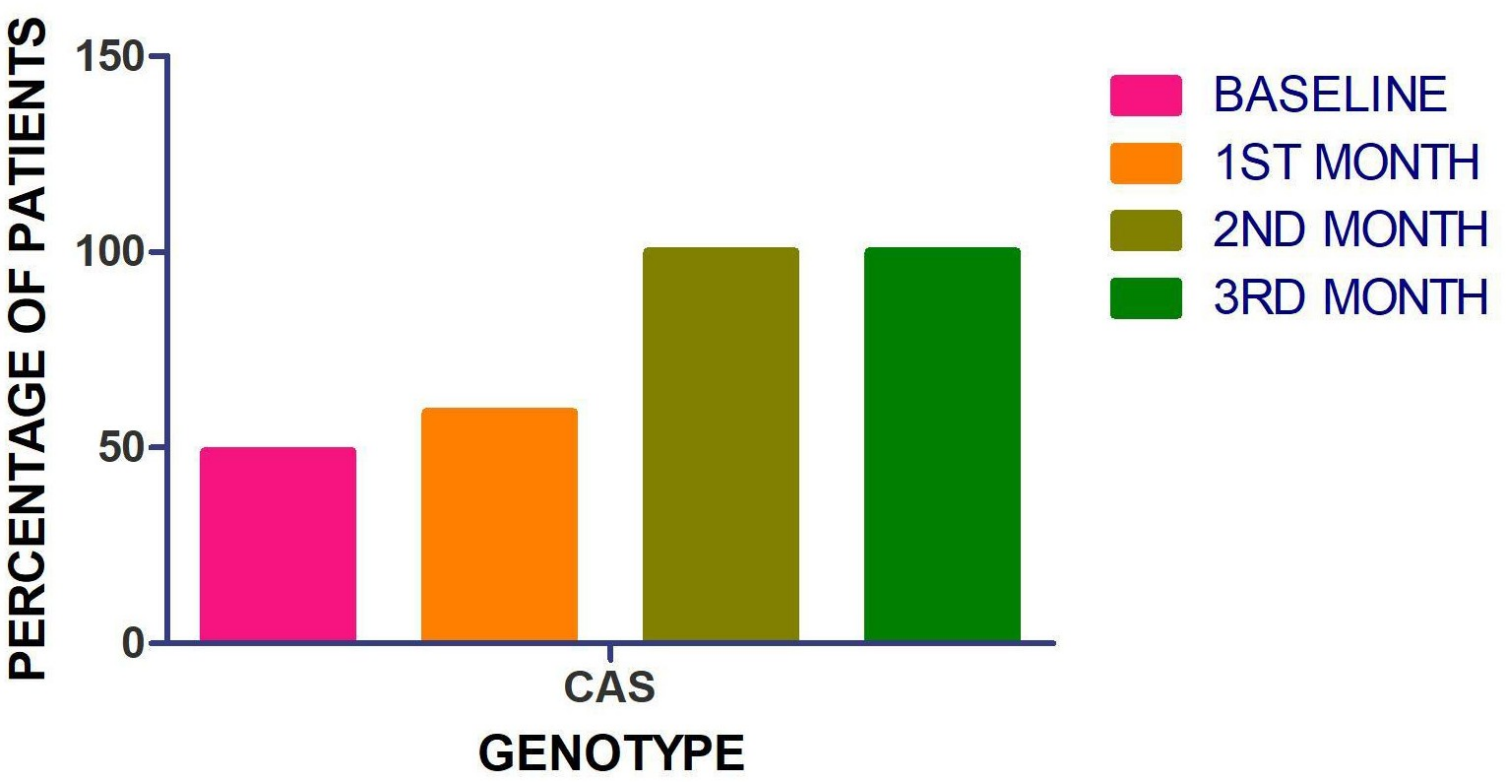
Detection of CAS genotype in serial follow up cultures from category I patients at different time points (Baseline, 1st, 2nd and 3^rd^ month).

The majority of patients harboring polyclonal infections and exhibiting drug resistance belonged to the Cas1_Delhi lineage. CAS lineage contributed to 49% of the baseline cultures, increasing to 59% of the first month cultures and 100% of the 2^nd^ and 3^rd^ month cultures (though the number of cultures growing in the 2^nd^ and 3^rd^ month were small) [Fig 4]. CAS lineage also contributed to 71% of the baseline cultures resistant to INH and 5/6 MDR isolates (83.3%) were CAS spoligotype, while 1(16.7%) was Beijing. [S6 Table]

### Patients with recurrence

Eight patients had recurrence, between 8 to 11 months. Six patients had genotype CAS1 at the baseline (two along with EAI), four relapsed with CAS1 genotype, and 1 had recurrence with Manu2 while one patient was diagnosed clinically but did not grow culture during recurrence. Two other patients who had T2 and Orphan genotypes at the baseline now had CAS1 genotype. The only change in the sensitivity pattern was in the patient who had recurrence with Manu2, which now showed a phenotype resistant to Ofloxacin. Five of eight patients were resistant to INH at baseline. [Table 2]

**Table 2:** Recurrent TB cases: Genotype at baseline and during recurrent episode in cured Rifampicin sensitive TB patients.

PATIENT DETAILS/SMEAR DETAILS	BASELINE CULTURE SPOLIGOTYPE /DST(R/I/O/K)	BASELINE TLA SPOLIGOTYPE	RECURRENT EPISODE(MONTH) SPOLIGOTYPE/DST (R/I/O/K)
cat I2142A2 2+	CAS1-DELHI/SSSS	CAS1_DELHI(1–5)	11^TH^ MONTH MANU2(1–5) SSRS
cat I6911 3+	CAS1/SSSS	CAS1_DELHI(1–2); EAI_SOM(3–4)	10^TH^ MONTH CAS1_DELHI(1–5) SSSS
cat I3554 2+	CAS1_DELHI/SSSS	CAS1_DELHI(1–3); EAI5(4)	10^TH^ MONTH CAS1_DELHI(1–6) SSSS
cat I0879 1+	ORPHAN/SRSS	CAS(1–3)	8^TH^ MONTH CAS1_DELHI(1–4) SRSS
cat I4991 2+	CAS1_DELHI/SRSS	CAS1_DELHI(1–5)	8TH MONTH CAS1_DELHI(1–4) SRSS
cat I2519 1+	T2/SRSS	T2(1–4)	10TH MONTH CAS1_DELHI(1–4) H1(5) SRSS
cat I3165 1+	CAS1_DELHI/SRSS	CAS1_DELHI(1–3)	8^TH^ MONTH CULTURE NEGATIVE
cat I2108 1+	CAS1_DELHI/SRSS	CAS1_DELHI(1–4)	9^TH^ MONTH CAS1_DELHI(1–4) SSSS

## Discussion

The classical teaching suggests that TB disease is the result of a single infecting Mtb genotype, which confers immunity from further infections. However, polyclonal infections have been reported in TB, mostly in high incidence settings, jails and hospitals with reports from crowded prisons in Georgia and from South Africa.[30] Mixed infections are reported in as many as 10–20% cases [3, 10–13]. In the present study, we tried to explore the prevalence of mixed infection at the beginning and through the treatment of TB.

Despite advances in strain genotyping, the limit of detection and resolution of the available techniques would compromise the detection of mixed infections.[3, 8, 31–32] Conventionally, mixed infections were detected using genotypic methods that have limited sensitivity because of the limited numbers of markers used to distinguish strains. [33,34] Shamputa et al reported increasing number of sputum samples improved likelihood of mixed infections detection.[32] Barczak et al reported that all pulmonary lesions may not be open to the airways and sputum may not adequately reflect the heterogeneity in underlying infections.[35] Mallard et al., 2010 and Richardson et al., 2002 reported that the timing of sputum affects the sensitivity of mixed infection detection. [36–37] Bates et al., 1976; Chaves et al., 1999; du Plessis et al., 2001; Kong et al., 2007 suggested that specimens sampled from multiple sites increases the likelihood of mixed infection detection, it could also be associated to organ tropism shown by some lineages. [38–41] Huang et al., 2010; Lew et al., 2008 successfully documented that mixed infections could be detected directly using lineage specific PCR on samples. [12, 42] However, Martín et al., 2010 found that the clonal composition changes after culture.[43]

MIRU-VNTR is currently the most widely used method for the detection of mixed infections; however its sensitivity is limited by set of loci used. [44] Another limitation of the method is difficulty in drawing distinctions between clonal heterogeneity and mixed infections. [27, 45] Perez-Lago et al, 2011 demonstrated microevolution in 12% isolates while studying disease transmission. [46] Studies have also demonstrated microevolution within patients or during transmission, using WGS. [47] In the current study, we additionally used SNP genotyping method to detect mixed infections. Three cultures appeared to have microevolution events.

Use of single colony genotyping was helpful in not only detecting different clones of mycobacteria present in the sample, but also due to quicker growth of colonies on TLA media (Middlebrook 7H11), detected mixed infection early. This method was most sensitive and helped detect six more samples with mixed infections over those detected by MIRU-VNTR typing and SNP genotyping.

Our study has brought forth the prevalence of mixed infection in a large number of patients with no past history of TB. These patients were initiated on standard of care regimen for Rifampicin sensitive TB and followed up. The follow-up cultures were positive in 37/133(27.8%) patients at different time points. Twenty-four of 37 (64.9%) patients with serial culture positives showed different genotypes at different time points. Cultures done on TLA (7H11) picked up more mixed infections during follow-up. The prevalence of mixed infections at baseline has been reported before, but the presence of mixed infection at later time points while the patient is on treatment is being reported for the first time in this study. The change in the genotype while on treatment is a very important new observation. This indicates the presence of multiple clones with predominance of a few at different points in time. The response of the different clones to the ATT may vary and hence the likelihood of finding a new clone in the same patient during follow-up could be explained.

Six of these 24 patients with change of genotype in serial LJ cultures also showed different drug susceptibility patterns in the follow-up cultures. Four isolates gained resistance to INH, while two lost resistance. Gain of resistance, midway through ongoing treatment may lead to treatment failure or relapse. These findings could have a bearing on the treatment of patients. Most high incidence countries are not equipped to conduct drug susceptibility testing at the baseline, repeating the DST during follow-up could be further more difficult.

Nathavitharana et al 2017 demonstrated polyclonal TB primarily in drug resistant TB. [48] Our observations of statistically significant association of drug resistance with polyclonality reiterate the same findings. CAS lineage contributed to nearly half of the baseline cultures and more of the subsequently grown cultures. Nearly 70% of INH resistant baseline cultures belonged to CAS lineage. These findings not only indicate that CAS lineage may contribute towards polyclonality and drug resistance but also that this lineage may take longer to respond to standard treatment regimen. However, this further needs to be elucidated in larger cohorts.

Eight patients had recurrent TB, between 8 to 11 months. Five patients were resistant to INH at baseline. Six patients had genotype CAS1 at the baseline (two with EAI) and four relapsed with CAS1 genotype, while 1 now had Manu2 and another one did not grow culture during recurrence though he was clinically diagnosed as a relapse. Two other patients, who had T2 and Orphan genotypes at the baseline, now had CAS1 genotype. The only change in the sensitivity pattern was in the patient who had recurrence with Manu2, now showed a phenotype resistant to ofloxacin. Guerra Assunção et al 2014 have reported association of relapses with INH resistance and with Lineage 3(CAS). [49]

Polyclonal Mtb infections may have a negative impact on drug resistance testing performed by both phenotypic [e.g., proportion method) and genotypic methods (e.g., GeneXpert MTB/RIF). [10, 50] Hetero-resistance, which is primarily the result of mixed infections, would hence be missed.[51] Van Rie et al have reported that mixed infections with strains of different resistance phenotypes compromises treatment outcomes using standard combination treatment regimens. [52] Some studies from South Africa have reported that unmasking of the MDR strains during treatment poses great threat to the national programs where a patient would be misclassified to be harboring drug sensitive strain and be treated accordingly. [53–54]

Use of Whole Genome Sequencing could have added to the value of the study. Sobkowiak et al 2018 used WGS to demonstrate mixed infection in 10% patients in Malawi. [55] Mortensen et al, 2016 found the utility of WGS in tracing transmission and identifying micro-epidemics in Greenland. [56] WGS has been reported primarily for studying disease epidemiology, transmission and resistance. However, due to the high expense involved, current study was built on the collective outcome of three efficient typing methods. [57]

The study findings have an important bearing on the current understanding of disease pathogenesis. The classical teaching of single genotypes being responsible for disease is put to question by these observations. The fact that 43% of the infections were detected as having more than one genotypes being present in the tubercular cavity clearly gives credence to the hypothesis that polyclonal infections may in fact be the rule rather than the exception. The rest of the samples may have escaped detection due to lower bacillary load or the limit of detection of culture and typing methods.

Further, the concepts of Reinfection and Reactivation may be open to question, looking at the evidence found in the current study. Additional, carefully designed studies may contribute to the data available on mixed infections.

## Supporting information

S1 Fig UPGMA-dendrogram based on MIRU-VNTR and spoligotype pattern of 133 samples under study. (DOCX)

S2 Fig A radial tree illustrating evolutionary relationships among different MTB spoligotypes of the study. (DOCX)

S1 Table Stratification of clinical presentation of disease and socio-demographic characteristics of patients. (DOCX)

S2 Table Drug resistance pattern of study participants based on liquid DST [LC-DST] on MGIT- 960. (DOCX)

S3 Table Frequencies of major spoligotypes /lineages of 133 culture positive isolates classified by SPOLDB4.0. (DOCX)

S4 Table Detailed genotyping results of orphan strains (n = 13) and their corresponding spoligotyping, MIRU-VNTR and SNP genotyping recorded among a 133 M. tuberculosis strains from patients. (DOCX)

S5 Table Determination of mixed infection by comparing different genotyping methods. (DOCX)

S6 Table Distribution of the CAS genotype in serial follow up cultures of category I patients. (DOCX)
