# Supplementary material for: Mycobacterium tuberculosis polyclonal infections through treatment and recurrence
Source: PLoS One. 2020 Aug 19;15(8):e0237345. doi: 10.1371/journal.pone.0237345 (PMC7437862; doi:10.1371/journal.pone.0237345)

S1 Fig: UPGMA-dendrogram based on MIRU-VNTR and spoligotype pattern of 133 samples under study. The lineage listed corresponds to the designation of spoligotypes as used in the SpolDB4 database. MIRU-VNTR patterns for the 24 loci andspoligotypes for 133 isolates are displayed in each line. The columns correspond to the MIRU-VNTR loci in the following order: MIRU02, VNTR0424, ETR C, MIRU04, MIRU40, MIRU10, MIRU16, MIRU20, VNTR1955, MIRU23, MIRU24; MIRU26, MIRU27, MIRU31, MIRU39, ETR A, QUB-11b, VNTR2347, VNTR3171, QUB-26, VNTR2401, VNTR3690, VNTR4156, and ETR B. Different colours are used to denote each lineage. BEI: green, CAS: red

- Crimson, EAI: yellow, H: light blue, MANU1: indigo, MANU2: brown, Orphan: violet, T: dark orchid, X: deep blue. The tree was calculated by using the MIRU-VNTR plus website.


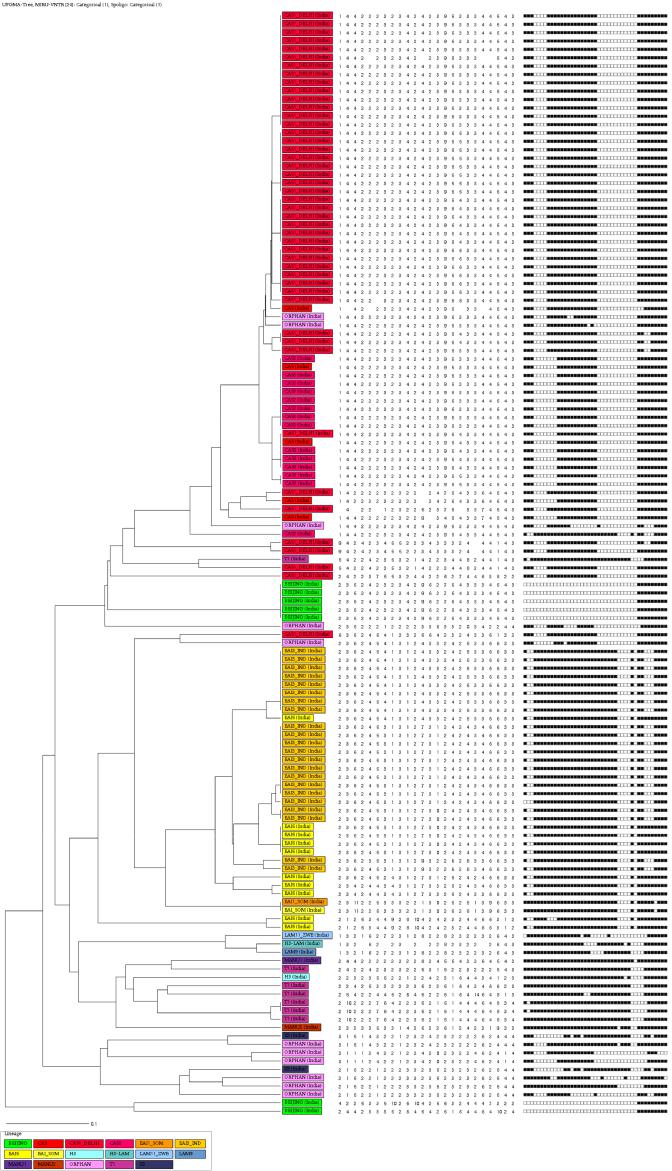

Supplement: S1 Fig — (DOCX) [file pone.0237345.s001.docx]
