# Supplementary material for: Mycobacterium tuberculosis polyclonal infections through treatment and recurrence
Source: PLoS One. 2020 Aug 19;15(8):e0237345. doi: 10.1371/journal.pone.0237345 (PMC7437862; doi:10.1371/journal.pone.0237345)

S2 Fig:A radial tree illustrating evolutionary relationships among different MTB spoligotypes of the study**.** The radial neighbor-joining tree is based on 24 loci MIRU-VNTR and 43 spacer spoligotyping; showing the phylogenetic relationship of different strains in the study. Different colours are used to denote each lineage. BEI: green, CAS: red & Crimson, EAI: yellow, H: light blue, MANU1: indigo, MANU2: brown, Orphan: violet, T: dark orchid, X: deep blue. The tree was calculated by using the MIRU-VNTR plus website.


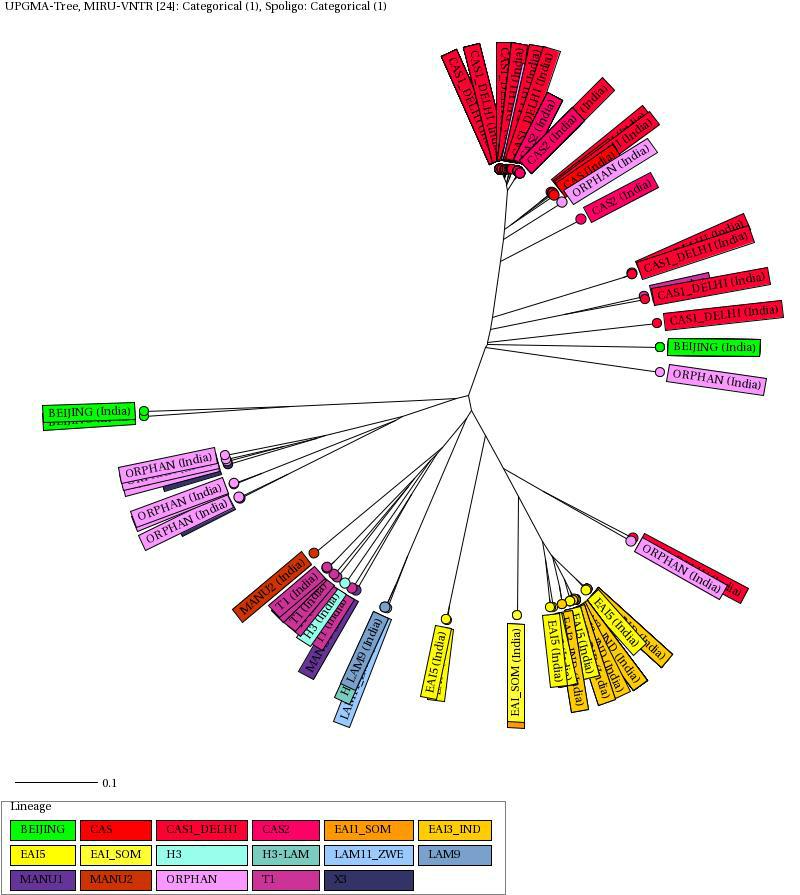

Supplement: S2 Fig — (DOCX) [file pone.0237345.s002.docx]
