# Supplementary material for: Mycobacterium tuberculosis polyclonal infections through treatment and recurrence
Source: PLoS One. 2020 Aug 19;15(8):e0237345. doi: 10.1371/journal.pone.0237345 (PMC7437862; doi:10.1371/journal.pone.0237345)
Supplement: S1 Table — (DOCX) [file pone.0237345.s003.docx]

S 1 Table: Stratification of clinical presentation of disease and socio-demographic characteristics of patients.

| **Characteristics** |  | **No. of cases** | **Sputum smear positive** | **Sputum smear negative** |
| --- | --- | --- | --- | --- |
| Sex | Female | 51 | 44 | 7 |
|  | Male | 106 | 94 | 12 |
| Address | Village | 19 | 18 | 1 |
|  | City | 138 | 120 | 18 |
| Family TB history | No | 33 | 28 | 5 |
|  | Yes | 124 | 110 | 14 |
| Smoker | No | 82 | 81 | 1 |
|  | Yes | 75 | 57 | 18 |
| Alcohol | No | 39 | 39 | 0 |
|  | Yes | 118 | 99 | 19 |
| BCG | Yes | 87 | 78 | 9 |
|  | No | 70 | 60 | 10 |
