# Supplementary material for: Mycobacterium tuberculosis polyclonal infections through treatment and recurrence
Source: PLoS One. 2020 Aug 19;15(8):e0237345. doi: 10.1371/journal.pone.0237345 (PMC7437862; doi:10.1371/journal.pone.0237345)
Supplement: S2 Table — (DOCX) [file pone.0237345.s004.docx]

S 2 Table: Drug resistance pattern of study participants based on liquid DST [LC-DST] on MGIT- 960.

| **Drug resistance** | **Resistance [n]** | **%** |
| --- | --- | --- |
| INH mono-resistant | 24 | 18% |
| Resistant to two drugs | 3 | 2.3% |
| Multi drug resistant | 6 | 4.5% |
| Pan-sensitive | 100 | 75.2% |
| Total | 133 | 100% |
