# Supplementary material for: Mycobacterium tuberculosis polyclonal infections through treatment and recurrence
Source: PLoS One. 2020 Aug 19;15(8):e0237345. doi: 10.1371/journal.pone.0237345 (PMC7437862; doi:10.1371/journal.pone.0237345)
Supplement: S3 Table — (DOCX) [file pone.0237345.s005.docx]

S 3 Table: Frequencies of major spoligotypes /lineages of 133 Culture positive isolates classified by SPOLDB4.0.

| **ST** |  | **CLADE** |  | **Number** | **of** |
| --- | --- | --- | --- | --- | --- |
|  |  |  |  | **isolates[n]** |  |
|  |  |  |  |  |  |
| ST 26 |  | CAS1_DEL | ◼◼◼🞏🞏🞏🞏◼◼◼◼◼◼◼◼◼◼◼◼◼◼◼🞏🞏🞏🞏🞏🞏🞏🞏🞏🞏🞏🞏◼◼◼◼◼◼◼◼◼ | 41 |  |
| ST288 |  | CAS2 | ◼◼◼🞏🞏🞏🞏🞏🞏🞏◼◼◼◼◼◼◼◼◼◼◼◼🞏🞏🞏🞏🞏🞏🞏🞏🞏🞏🞏🞏◼◼◼◼◼◼◼◼◼ | 18 |  |
| ST 25 |  | CAS1_DEL | ◼◼◼🞏🞏🞏🞏◼◼◼◼◼◼◼◼◼◼◼◼◼◼◼🞏🞏🞏🞏🞏🞏🞏🞏🞏🞏🞏🞏◼◼🞏🞏◼◼◼◼◼ | 5 |  |
| ST 357 |  | CAS | ◼◼◼🞏🞏🞏🞏◼◼◼◼◼◼◼◼◼◼◼◼◼◼◼🞏🞏🞏🞏🞏🞏🞏🞏🞏🞏🞏🞏🞏🞏◼◼◼◼◼◼◼ | 1 |  |
| ST 11 |  | EAI3_IND | ◼🞏🞏◼◼◼◼◼◼◼◼◼◼◼◼◼◼◼◼◼◼◼◼◼◼◼◼◼🞏🞏🞏🞏◼🞏◼◼🞏🞏🞏◼◼◼◼ | 22 |  |
| ST 48 |  | EAI_SOM | ◼◼◼◼◼◼◼◼◼◼◼◼◼◼◼◼◼◼◼◼◼◼◼◼◼◼◼◼🞏🞏🞏🞏◼🞏◼◼◼◼◼🞏◼◼◼ | 2 |  |
| ST 126 |  | EAI5 | ◼🞏🞏◼◼◼◼◼◼◼◼◼◼◼◼◼◼◼◼◼◼◼◼◼◼◼◼◼🞏🞏🞏🞏◼🞏◼◼◼◼◼◼◼◼◼ | 9 |  |
| ST 340 |  | EAI5 | ◼🞏🞏◼◼◼◼🞏🞏🞏◼◼◼◼◼◼◼◼◼◼◼◼◼◼◼◼◼◼🞏🞏🞏🞏◼🞏◼◼◼◼◼◼◼◼◼ | 1 |  |
| ST100 |  | MANU1 | ◼◼◼◼◼◼◼◼◼◼◼◼◼◼◼◼◼◼◼◼◼◼◼◼◼◼◼◼◼◼◼◼◼🞏◼◼◼◼◼◼◼◼◼ | 1 |  |
| ST1094 |  | MANU2 | ◼◼◼◼◼◼◼◼◼◼◼◼◼◼◼◼◼◼◼◼◼◼◼◼◼◼◼◼🞏◼◼🞏🞏◼◼◼◼◼◼◼◼◼◼ | 1 |  |
| ST 53 |  | T1 | ◼◼◼◼◼◼◼◼◼◼◼◼◼◼◼◼◼◼◼◼◼◼◼◼◼◼◼◼◼◼◼◼🞏🞏🞏🞏◼◼◼◼◼◼◼ | 3 |  |
| ST334 |  | T1 | ◼🞏◼◼◼◼◼◼◼◼◼◼◼◼◼◼◼◼◼◼◼◼◼◼◼◼◼◼◼◼◼◼🞏🞏🞏🞏◼◼◼◼◼◼◼ | 1 |  |
| ST 1252 |  | T1 | ◼◼◼◼◼◼◼◼◼◼◼◼◼◼◼◼◼◼🞏🞏◼◼◼◼◼◼◼◼◼◼◼◼🞏🞏🞏🞏◼◼◼◼◼◼◼ | 1 |  |
| UNKNOWN |  | T1 | ◼🞏◼◼◼◼◼◼◼◼◼◼◼◼◼◼◼◼◼◼◼◼◼◼◼◼◼◼◼◼◼◼🞏🞏🞏🞏◼◼◼◼◼◼◼ | 2 |  |
| ST 42 |  | LAM 9 | ◼◼◼◼◼◼◼◼◼◼◼◼◼◼◼◼◼◼◼◼🞏🞏🞏🞏◼◼◼◼◼◼◼◼🞏🞏🞏🞏◼◼◼◼◼◼◼ | 1 |  |
| UNKNOWN |  | LAM11_ZWE | 🞏◼◼◼◼◼◼◼◼◼◼◼◼◼◼◼◼🞏◼◼🞏🞏🞏🞏🞏◼🞏🞏🞏🞏🞏🞏🞏🞏🞏🞏🞏◼◼◼◼◼◼ | 1 |  |
| ST67 |  | H3_LAM | ◼◼◼◼◼◼◼◼◼◼◼◼◼◼◼◼◼◼🞏🞏🞏🞏◼◼◼◼◼◼◼◼🞏◼🞏🞏🞏🞏◼◼◼◼◼◼◼ | 1 |  |
| ST 50 |  | H3 | ◼◼◼◼◼◼◼◼◼◼◼◼◼◼◼◼◼◼◼◼◼◼◼◼◼◼◼◼◼◼🞏◼🞏🞏🞏🞏◼◼◼◼◼◼◼ | 1 |  |
| ST 1 |  | BEIJING | 🞏🞏🞏🞏🞏🞏🞏🞏🞏🞏🞏🞏🞏🞏🞏🞏🞏🞏🞏🞏🞏🞏🞏🞏🞏🞏🞏🞏🞏🞏🞏🞏🞏🞏◼◼◼◼◼◼◼◼◼ | 7 |  |
| ST 1315 |  | X3 | ◼◼◼🞏🞏🞏🞏🞏🞏🞏🞏🞏◼◼🞏◼◼🞏◼◼◼◼◼◼◼◼◼◼◼◼◼◼🞏🞏🞏🞏◼◼🞏◼◼◼◼ | 1 |  |
| OR 1 |  | ORPHAN | ◼◼◼🞏🞏🞏🞏◼◼◼◼◼◼🞏🞏◼◼◼◼◼◼🞏🞏🞏🞏🞏🞏🞏🞏🞏🞏🞏🞏◼◼◼◼◼◼◼◼◼◼ | 1 |  |
| OR2 |  | ORPHAN | ◼◼◼🞏🞏🞏🞏◼◼◼◼◼◼◼◼◼◼◼◼🞏◼🞏🞏🞏🞏🞏🞏🞏🞏🞏🞏🞏🞏🞏◼◼◼◼◼◼◼◼◼ | 1 |  |
| OR3 |  | ORPHAN | ◼◼◼🞏🞏🞏🞏◼◼◼◼◼◼◼🞏🞏🞏🞏🞏🞏🞏🞏◼🞏🞏🞏🞏🞏🞏🞏🞏🞏🞏◼◼🞏🞏🞏🞏🞏◼◼◼ | 1 |  |
| OR4 |  | ORPHAN | ◼◼◼🞏🞏🞏🞏◼◼◼◼◼◼◼◼◼◼◼◼◼◼◼◼◼◼◼◼◼🞏◼◼◼🞏🞏◼◼◼◼◼◼◼◼◼ | 1 |  |
| OR5 |  | ORPHAN | ◼◼◼🞏🞏🞏🞏◼◼◼◼◼◼◼🞏🞏🞏🞏🞏🞏🞏◼🞏🞏🞏🞏🞏🞏🞏🞏🞏🞏🞏🞏◼◼◼🞏🞏◼◼◼◼ | 1 |  |
| OR6 |  | ORPHAN | ◼◼◼◼◼◼◼◼🞏🞏◼◼◼◼◼◼◼◼◼◼🞏🞏🞏◼🞏🞏🞏🞏🞏🞏◼🞏🞏🞏🞏◼◼◼◼◼◼◼🞏 | 1 |  |
| OR7 |  | ORPHAN | ◼◼◼🞏🞏🞏🞏◼◼◼◼◼◼◼◼◼◼◼◼◼◼🞏🞏🞏🞏🞏🞏🞏🞏🞏🞏🞏🞏🞏🞏🞏🞏◼◼◼🞏🞏🞏 | 1 |  |
| OR8 |  | ORPHAN | ◼◼◼◼◼◼◼◼◼◼◼◼◼◼◼◼◼◼◼◼◼◼◼◼◼◼◼◼🞏🞏🞏🞏🞏🞏🞏🞏◼◼◼◼◼◼◼ | 1 |  |
| OR9 |  | ORPHAN | ◼◼◼🞏🞏🞏🞏◼◼◼◼◼🞏🞏🞏🞏◼◼◼◼◼🞏🞏🞏🞏🞏🞏🞏🞏🞏🞏🞏🞏🞏◼◼◼◼◼◼◼◼◼ | 1 |  |
| OR10 |  | ORPHAN | ◼◼◼🞏🞏🞏🞏🞏🞏🞏◼◼◼◼◼◼◼◼◼◼◼◼🞏🞏🞏🞏🞏🞏🞏🞏🞏🞏🞏🞏🞏🞏🞏🞏◼◼◼◼◼ | 1 |  |
| OR11 |  | ORPHAN | ◼◼◼🞏🞏🞏🞏◼◼◼🞏◼◼◼🞏🞏🞏🞏🞏🞏🞏◼🞏🞏🞏🞏🞏🞏🞏🞏🞏🞏🞏🞏◼◼◼🞏🞏◼◼◼◼ | 1 |  |
| OR12 |  | ORPHAN | ◼◼◼🞏🞏🞏🞏◼◼🞏🞏◼◼◼🞏🞏🞏🞏🞏🞏🞏◼🞏🞏🞏🞏🞏🞏🞏🞏🞏🞏🞏🞏◼◼◼🞏🞏◼◼◼◼ | 1 |  |
| OR13 |  | ORPHAN | ◼◼◼◼◼◼◼◼◼◼◼◼◼◼◼◼◼◼◼◼◼◼◼◼◼◼◼◼🞏🞏🞏🞏🞏🞏🞏🞏◼◼◼◼◼🞏◼ | 1 |  |
