# Supplementary material for: Mycobacterium tuberculosis polyclonal infections through treatment and recurrence
Source: PLoS One. 2020 Aug 19;15(8):e0237345. doi: 10.1371/journal.pone.0237345 (PMC7437862; doi:10.1371/journal.pone.0237345)
Supplement: S4 Table — (DOCX) [file pone.0237345.s006.docx]

S 4 Table: Detailed genotyping results of Orphan strains (n = 13) and their corresponding spoligotyping, MIRU-VNTR and SNP genotyping recorded among a 133 M. tuberculosis strains from patients.

| **S.NO** | **SPOLIGOTYPE DESCRIPTION** | **MIRU-VNTR** | **REAL TIME BASED SNP GENOTYPING** | | |  |  |  |  |
| --- | --- | --- | --- | --- | --- | --- | --- | --- | --- |
|  |  |  |  |  |  |  |  |  |  |
|  |  |  | **katG463** | **Rv2952** |  | **RV3221** | **Rv3804** | **M. Africanum** | **M. Africanum** |
|  |  |  |  |  |  |  |  | **West-African 2** | **West-African 1** |
|  |  |  |  |  |  |  |  |  |  |
| OR 1 | 1110000111111001111110000000000001111111111 | 236246413124332425336633 | NEGATIVE | NEGATIVE |  | NEGATIVE | NEGATIVE | NEGATIVE | NEGATIVE |
| OR2 | 1110000111111111111010000000000000111111111 | 144222323424239633344543 | NEGATIVE | NEGATIVE |  | NEGATIVE | POSITIVE | NEGATIVE | NEGATIVE |
| OR3 | 1110000111111100000000100000000001100000111 | 144222323424239633344543 | NEGATIVE | NEGATIVE |  | NEGATIVE | NEGATIVE | NEGATIVE | NEGATIVE |
| OR4 | 1110000111111111111111111111011100111111111 | 144222323424239633344643 | NEGATIVE | NEGATIVE |  | NEGATIVE | NEGATIVE | NEGATIVE | NEGATIVE |
|  |  |  |  |  |  |  |  |  |  |
| OR5 | 1110000111111100000001000000000000111001111 | 224535591370166124441624 | NEGATIVE | NEGATIVE |  | NEGATIVE | NEGATIVE | NEGATIVE | NEGATIVE |
| OR6 | 1111111100111111111100010000001000011111110 | 316143221232423222262444 | NEGATIVE | NEGATIVE |  | NEGATIVE | NEGATIVE | NEGATIVE | NEGATIVE |
| OR7 | 1110000111111111111110000000000000000111000 | 216221222332362323262644 | NEGATIVE | NEGATIVE |  | NEGATIVE | NEGATIVE | NEGATIVE | NEGATIVE |
| OR8 | 1111111111111111111111111111000000001111111 | 252235642545225174464834 | NEGATIVE | NEGATIVE |  | NEGATIVE | NEGATIVE | NEGATIVE | NEGATIVE |
| OR9 | 1110000111110000111110000000000000111111111 | 311124221234202322462414 | NEGATIVE | NEGATIVE |  | NEGATIVE | NEGATIVE | NEGATIVE | NEGATIVE |
| OR10 | 1110000000111111111111000000000000000011111 | 216221222332352323262544 | NEGATIVE | NEGATIVE |  | NEGATIVE | NEGATIVE | NEGATIVE | NEGATIVE |
| OR11 | 1110000111011100000001000000000000111001111 | 311242212342023224624144 | NEGATIVE | NEGATIVE |  | NEGATIVE | NEGATIVE | NEGATIVE | NEGATIVE |
| OR12 | 1110000110011100000001000000000000111001111 | 235222122223363320342244 | NEGATIVE | NEGATIVE |  | NEGATIVE | NEGATIVE | NEGATIVE | NEGATIVE |
| OR13 | 1111111111111111111111111111000000001111101 | 216221222332362323262644 | NEGATIVE | NEGATIVE |  | NEGATIVE | NEGATIVE | NEGATIVE | NEGATIVE |
