# Supplementary material for: Mycobacterium tuberculosis polyclonal infections through treatment and recurrence
Source: PLoS One. 2020 Aug 19;15(8):e0237345. doi: 10.1371/journal.pone.0237345 (PMC7437862; doi:10.1371/journal.pone.0237345)
Supplement: S5 Table — (DOCX) [file pone.0237345.s007.docx]

S 5 Table: Determination of mixed infection by comparing different genotyping methods.

| **Combined mixed infection detection** | **Mixed infection detection by utilizing 24-locus VNTR typing:** | | |  |
| --- | --- | --- | --- | --- |
| **by SNP typing and spoligotyping** |  |  |  |  |
|  |  |  |  |  |
| **Mixed infection** | **Double alleles at two** | **Double allele** | **All single** | **Total** |
|  | **or more loci** |  |  |  |
|  |  | **at one locus** | **alleles** |  |
|  |  |  |  |  |
| Present | 29 | 8 | 0 | 37 |
|  |  |  |  |  |
| Absent | 1 | 3 | 92 | 96 |
|  |  |  |  |  |
| Total | 30 | 11 | 92 | 133 |
|  |  |  |  |  |
