# Supplementary material for: Mycobacterium tuberculosis polyclonal infections through treatment and recurrence
Source: PLoS One. 2020 Aug 19;15(8):e0237345. doi: 10.1371/journal.pone.0237345 (PMC7437862; doi:10.1371/journal.pone.0237345)
Supplement: S6 Table — (DOCX) [file pone.0237345.s008.docx]

S6 Table: Distribution of the CAS genotype in serial follow up cultures of Category I patients.

| Genotype | **Baseline (n=133)** | **1^st^ month(n=22)** | **2^nd^ month(n=3)** | **3^rd^ month(n=2)** |
| --- | --- | --- | --- | --- |
| CAS | 65/133 | 13/22 | 3/3 | 2/2 |
|  | 48.9% | 59.1% | 100% | 100% |
